# The complete mitochondrial genome of *Chionoecetes japonicus* (Crustacea: Decapoda: Majoidea)

**DOI:** 10.1080/23802359.2020.1827061

**Published:** 2020-10-07

**Authors:** Yong Hwi Kim, Kang-Rae Kim, Jong Yeon Park, Mu Sung Sung, Bong Han Yun, Ahhyeon Jeon, Moo-Sang Kim, In-Chul Bang

**Affiliations:** a Department of Life Science and Biotechnology, Soonchunhyang University, Asan, Republic of Korea; b Department of Biology, Soonchunhyang University, Asan, Republic of Korea; c Department of Bioinformatics, MOAGEN, Daejeon, Republic of Korea

**Keywords:** Crustacea, Decapoda, Majoidea, *Chionoecetes japonicus*, mitogenome

## Abstract

The complete mitochondrial genome of *Chionoecetes japonicus* was sequenced using a specimen collected offshore in the East Sea. The genome includes 13 protein-coding genes (PCGs), 22 transfer RNA (*tRNA*) genes, two ribosomal RNA (*rRNA*) genes, and a control region (D-loop), with a total length of 16,060 bp. The overall nucleotide composition was 34.91% A, 17.29% C, 10.93% G, and 36.87% T, with 71.78% A + T. In the phylogenetic tree was constructed using maximum-likelihood and Bayesian inference analyses, *C. japonicus* and *C. japonicus pacificus* formed a genetic clade that was sister to *C. opilio*.

Red snow crab, *Chionoecetes japonicus* Rathbun, 1932, is found in the East Sea. Unlike *C*. *opilio*, which is found at 200–800 m, it can be observed at 500–2300 m (Park et al. 2003). The genus *Chionoecetes* is a very important fisheries resource, and its ecology and fishing are active research topics. However, for *Chionoecetes*, because only the mitochondrial genomes of *C. japonicus pacificus* and *C. opilio* are currently known, additional mitochondrial genomes need to be discovered to elucidate the systematic relationships among taxa in the genus.

The specimen used in this study was collected offshore of Ganggu, Yeongdeok-gun, the Republic of Korea on 1 June 2020 using a fish trap, fixed in 99.9% ethanol, and stored in the specimen storage facility of Soonchunhyang University (Voucher no. SUC19351). Genomic DNA was extracted from walking leg tissue using a HiGene^TM^ Genomic DNA Prep Kit (Biofact, Daejeon, Republic of Korea), and a qualified library was constructed by sequencing 2 × 150 bp paired-end reads on an MGISEQ-2000 platform (MGI Tech Co. Ltd, Shenzhen, China) to generate raw reads with a total of 8,831,783,100 bp (SRA accession no. SRR12462354).

The mitochondrial genome sequence was assembled using Geneious R11 software (Kearse et al. 2012) and a reference sequence (GenBank accession no. AB735678), by mapping reads against contigs with the Geneious mapper tool (settings: no gaps allowed, 3% maximum mismatch per reading, word length = 40). Annotations were performed with MITOS WebServer (Bernt et al. 2013) and corrected manually.

The mitochondrial genome of *C*. *japonicus* (GenBank accession no. MT750295) is 16,060 bp long and includes 13 protein-coding genes (PCGs), 22 transfer RNA (*tRNA*) genes, two ribosomal RNA (*rRNA*) genes, and a control region (D-loop). Four PCGs (*nd5*, *nd4*, *nd4L*, and *nd1*), eight *tRNA*s (*tRNA^His^*, *tRNA^Phe^*, *tRNA^Pro^*, *tRNA^Leu^*, *tRNA^Val^*, *tRNA^Gln^*, *tRNA^Cys^*, and *tRNA^Tyr^*), and two *rRNA*s (12S and 16S *rRNA*) are encoded on the light strand. The overall base composition is 34.91% A, 17.29% C, 10.93% G, and 36.87% T, with 71.78% A + T, which is similar to the base content and AT bias of mitochondrial genomes in other Crustacea (Xing et al. 2017; Lu et al. 2020; Wang et al. 2020).

Of the 13 PCGs, the start codon is ATG in six, ATT in three, and GTG in two; the start codons of the *nd4L* and *nd6* genes are ATA and ATC, respectively. Three of the PCGs terminate with incomplete stop codons, T (*nd5*, *nd4*, and *nd1*), and the remaining 10 end with complete stop codons (TAA or TAG).

In addition, as in previous studies, all genes located in the β-strand in the *Maja* species compared here formed a single block located between *tRNA^Glu^* and a control region. By contrast, in the genus *Chionoecetes*, the arrangements of the general mitochondrial genes and tRNAs were similar to those in Brachyura, such as in the genus *Damithrax* (Basso et al. 2017; Jeong et al. 2020).

All 13 PCGs on each mitochondrial genome used in the phylogenetic analysis were downloaded from the National Center for Biotechnology Information and subjected to an analysis based on the alignment results obtained with MAFFT 7.450 (Katoh et al. 2002; Katoh and Standley 2013). GTRGAMMA was found to be the optimal model based on the corrected Akaike information criterion (AICc) using jModelTest 2.1.10 (Guindon and Gascuel, 2003; Darriba et al., 2012). The maximum-likelihood (ML) tree was constructed with 1,000 bootstrap replications using PhyML 3.0 (Guindon et al. 2010), and the Bayesian inference (BI) tree was run for 1,000,000 generations using MrBayes 3.2.7 (Ronquist et al. 2012). In addition, two *Maja* species belonging to the superfamily Majoidea and one *Damithrax* species were used as the outgroup. The phylogenetic tree was constructed based on ML and BI analyses (Figure 1).

**Figure 1.**
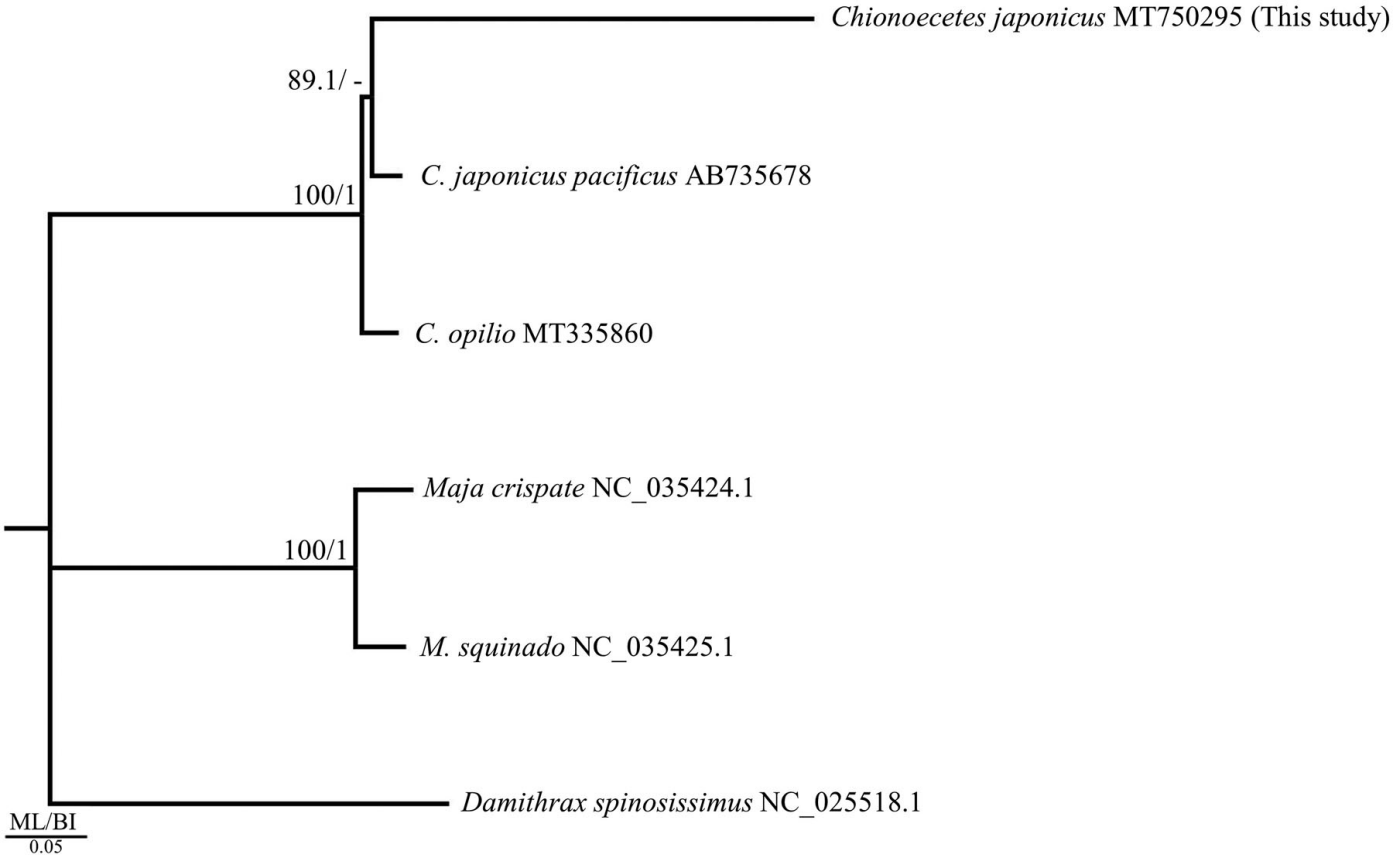
Phylogenetic tree of the genus *Chionoecetes* obtained from maximum-likelihood (ML) and Bayesian inference (BI) analyses of 13 protein-coding genes (PCGs). Bootstrap values above 80% in the ML analysis and posterior probabilities above 0.90 in the BI analysis are displayed at the base of each node. The best-fit evolutionary model was the GTRGAMMA model. The GenBank accession numbers are provided after each scientific name.

In the phylogenetic tree, two mitochondrial genomes (GenBank accession no. AB735378 and MT750295) from *C. japonicus* formed a clade sister to *C. opilio* (Figure 1). Similar results were observed for the *co1* gene in Azuma et al. (2011). Altogether, these and previous findings support the current taxonomic system.

The basic data on the complete mitochondrial genome of *C. japonicus* provided in this study will be an important resource for population genetic analysis, and will also be helpful for molecular phylogenetic studies of other species within the genus *Chionoecetes* that have not yet been discovered.

## Data Availability

The data that support the findings of this study are openly available in GenBank of NCBI at https://www.ncbi.nlm.nih.gov, reference number MT750295.

## References

[CIT0001] Azuma N, Grant WS, Templin WD, Kunihiro Y, Mihara E, Yanagimoto T, Abe S. 2011. Molecular phylogeny of a red-snow-crab species complex using mitochondrial and nuclear DNA markers. Zool Sci. 28(4):286–292. 10.2108/zsj.28.286 21466347

[CIT0002] Basso A, Babbucci M, Pauletto M, Riginella E, Patarnello T, Negrisolo E. 2017. The highly rearranged mitochondrial genomes of the crabs *Maja crispata* and *Maja squinado* (Majidae) and gene order evolution in Brachyura. Sci Rep. 7(1):1–17. 28642542 10.1038/s41598-017-04168-9 PMC5481413

[CIT0003] Bernt M, Donath A, Jühling F, Externbrink F, Florentz C, Fritzsch G, Pütz J, Middendorf M, Stadler PF. 2013. MITOS: Improved de novo metazoan mitochondrial genome annotation. Mol Phylogenet Evol. 69(2):313–319. 22982435 10.1016/j.ympev.2012.08.023

[CIT0004] Darriba D, Taboada GL, Doallo R, Posada D. 2012. jModelTest 2: more models, new heuristics and parallel computing. Nat Methods. 9(8):772. 10.1038/nmeth.2109 PMC4594756 22847109

[CIT0005] Guindon S, Gascuel O. 2003. A simple, fast and accurate method to estimate large phylogenies by maximum-likelihood. Syst Biol. 52(5):696–704. 14530136 10.1080/10635150390235520

[CIT0006] Guindon S, Dufayard JF, Lefort V, Anisimova M, Hordijk W, Gascuel O. 2010. New algorithms and methods to estimate maximum-likelihood phylogenies: assessing the performance of PhyML 3.0. Syst Biol. 59(3):307–321. 20525638 10.1093/sysbio/syq010

[CIT0007] Jeong JH, Ryu S, Kim W. 2020. The complete mitogenome of the *Chionoecetes opilio* (Crustacea: Decapoda: Oregoniidae) and its unique characteristics. Mitochondrial DNA Part B. 5(3):2550–2552. 33457859 10.1080/23802359.2020.1780974 PMC7782958

[CIT0008] Katoh K, Misawa K, Kuma KI, Miyata T. 2002. MAFFT: a novel method for rapid multiple sequence alignment based on fast Fourier transform. Nucleic Acids Res. 30(14):3059–3066. 12136088 10.1093/nar/gkf436 PMC135756

[CIT0009] Katoh K, Standley DM. 2013. MAFFT multiple sequence alignment software version 7: improvements in performance and usability. Mol Biol Evol. 30(4):772–780. 23329690 10.1093/molbev/mst010 PMC3603318

[CIT0010] Kearse M, Moir R, Wilson A, Stones-Havas S, Cheung M, Sturrock S, Buxton S, Cooper A, Markowitz S, Duran C, Thierer T, Ashton B, Meintjes P, Drummond A. 2012. Geneious Basic: An integrated and extendable desktop software platform for the organization and analysis of sequence data. Bioinformatics. 28(12):1647–1649. 22543367 10.1093/bioinformatics/bts199 PMC3371832

[CIT0011] Lu X, Gong L, Zhang Y, Chen J, Liu L, Jiang L, Lü Z, Liu B, Tong G, Wei X. 2020. The complete mitochondrial genome of *Calappa bilineata*: the first representative from the family Calappidae and its phylogenetic position within Brachyura. Genomics. 112(3):2516–2523. 32045669 10.1016/j.ygeno.2020.02.003

[CIT0012] Park JH, Min JG, Kim TJ, Kim JH. 2003. Comparison of food components between red-tanner crab, *Chionoecetes japonicus* and Neodo-Daege, a new species of *Chionoecetes* sp. caught in the East Sea of Korea. Korean J Fish Aquatic Sci. 36(1):62–64.

[CIT0013] Rathbun MJ. 1932. Preliminary descriptions of new species of Japanese crabs. Proc Biol Soc Wash. 45:29–38.

[CIT0014] Ronquist F, Teslenko M, van der Mark P, Ayres DL, Darling A, Höhna S, Larget B, Liu L, Suchard MA, Huelsenbeck JP. 2012. MrBayes 3.2: efficient bayesian phylogenetic inference and model choice across a large model space. Syst Biol. 61(3):539–542. 22357727 10.1093/sysbio/sys029 PMC3329765

[CIT0015] Wang Z, Shi X, Guo H, Tang D, Bai Y, Wang Z. 2020. Characterization of the complete mitochondrial genome of *Uca lacteus* and comparison with other Brachyuran crabs. Genomics. 112(1):10–19. 31175980 10.1016/j.ygeno.2019.06.004

[CIT0016] Xing Y, Zhou L, Hou Y, Wang X, Zhang C, Zhang H, Wang R, Pan D, Sun H. 2017. Complete mitochondrial genomes from two species of Chinese freshwater crabs of the genus *Sinopotamon* recovered using next-generation sequencing reveal a novel gene order (Brachyura, Potamidae). ZooKeys. 705:41–60. 10.3897/zookeys.705.11852 PMC5674035 29118611

